# Association between body weight misperception and dietary patterns in Brazilian adolescents: Cross-sectional study using ERICA data

**DOI:** 10.1371/journal.pone.0257603

**Published:** 2021-09-23

**Authors:** Simoni Urbano da Silva, Mariane de Almeida Alves, Francisco de Assis Guedes de Vasconcelos, Vivian Siqueira Santos Gonçalves, Laura Augusta Barufaldi, Kenia Mara Baiocchi de Carvalho

**Affiliations:** 1 Faculty of Health Sciences, Graduate Program of Public Health, University of Brasilia, Brasilia, Federal District, Brazil; 2 Department of Nutrition, School of Public Health, University of Sao Paulo, Sao Paulo, São Paulo, Brazil; 3 Graduate Program in Nutrition, Federal University of Santa Catarina, Florianópolis, State of Santa Catarina, Brazil; 4 Population Research Division, Brazilian National Cancer Institute José Alencar Gomes da Silva, Rio de Janeiro, Rio de Janeiro, Brazil; Instituto Federal Goiano, BRAZIL

## Abstract

The association between body image and eating behaviors or weight control strategies has been demonstrated in the scientific literature, but there is a lack of evidence on the association between weight misperception and food consumption indicators in adolescents. This study aimed to evaluate the association between weight misperception and dietary patterns (DPs) in the Brazilian Study of Cardiovascular Risks in Adolescents (ERICA). It was a national school-based cross-sectional study conducted among students aged 12–17 years. Data were collected in the form of anthropometric measurements, responses in self-answered questionnaires, and 24-h dietary recall. The following variables were assessed: weight underestimation and overestimation (independent variables), which were defined as the presence of a disagreement between nutritional status and self-perceived weight; dietary patterns (dependent variables), defined by a *posteriori* method using principal component factor analysis; and individual and demographic variables (covariates). Data analysis was performed using the Poisson regression models method, stratified by sex. A total of 52,038 adolescents with normal weights were evaluated. The weight misperception prevalence was 34.0% (95% confidence interval [CI]: 33.0, 35.0). Three DPs were identified: “Traditional Brazilian,” “Processed meat sandwiches and coffee,” and “Ultra-processed and sweet foods.” In girls, weight underestimation was directly associated with the “Traditional Brazilian” (1.24; 95% CI: 1.08, 1.43) and “Ultra-processed and sweet foods” DPs (1.29; 95% CI: 1.09, 1.54), and overestimation was inversely associated with all the DPs. In boys, a direct association between underestimation and the “Ultra-processed and sweet foods” DP (1.29; 95% CI: 1.10, 1.51) was found. Overestimation was inversely associated with the “Traditional Brazilian” DP (0.79; 95% CI: 0.63, 0.99). The inverse association between overestimation and the “Traditional Brazilian” DP, and the direct association between underestimation and the “Ultra-processed and sweet foods” DP indicated that weight misperception was related to unhealthy eating habits in Brazilian adolescents.

## Introduction

Adolescence is the phase of life between childhood and adulthood. According to the World Health Organization, adolescents are individuals in the 10 to 19 years age group. Adolescence is an important phase for the foundations of good health [1]. This phase is a period of rapid physiological, hormonal and behavioral changes, and is a critical stage to body image development, that is, the self-perception of one’s physical appearance or mental representation of one’s own body [2]. Body image is influenced by many factors, such as the opinions and perceptions of parents and friends [3,4], as well as media exposure through television, movies, magazines and virtual social networks [3,5–8].

The body image perception in adolescence may affect physical and mental health [5], especially as this perception may be distorted in relation to nutritional status [9–11]. Weight misperception is the disagreement between nutritional status and body weight perception [12,13], and in adolescents, it is associated to eating disorders, restrictive diets, low self-esteem, and higher weight gain in adulthood [11,13,14]. Both weight overestimation, when adolescents perceive themselves to be heavier than they are, and underestimation, when they perceive themselves to be lighter than their current weight, are associated with risk behaviors [9,15]. According to the first edition of the National Survey of School Health (PeNSE, in Portuguese), collected in Brazil in 2009, 25.1% of adolescents who were underweight overestimated their body weight and 49.4% of those who were overweight underestimated their weight. Among students with normal weight, 65.7% perceived their weight accurately [16].

Body dissatisfaction is a predictor of disordered eating behaviors. Initially adolescents may choose to diet or restrict food intake in attempts to change weight. Subsequently, they can develop bolder methods to control weight such as purging [17]. Worldwide, there is moderate evidence of an association between overestimation of weight and unhealthy weight control strategies (use of diet medication, purging, or fasting) and disordered eating behaviors in children and adolescents [18]. In Brazil, a direct relationship was found between weight concerns and food restriction [19] or other eating disorders [20] in adolescents. Further, among Brazilian adolescents, the perceived weight, rather than actual nutritional status, tended to be more associated with extreme efforts toward weight control such as vomiting or laxative use [21].

Due to rapid growth in adolescence, adequate nutrition is important for the achievement of full growth potential [22]. However, adolescents frequently restrict their calorie intake as a weight control strategy [23]. This behavior leads to an insufficient intake of nutrients and represents a risk factor to eating disorders such as anorexia and bulimia [24,25]. Excessive consumption of calories in adolescence is also common and is related to high intake levels of fast foods, candy, soft drinks and ultra-processed foods [25]. The intake of these products among adolescents is on the rise in Brazil [26], and is associated with a higher prevalence of obesity in the country [27]. It is estimated that between 1975 and 2016, the prevalence of obesity among children and adolescents, worldwide, increased from 0.7% to 5.6% in girls and from 0.9% to 7.8% in boys [28]. The corresponding data in Brazil are similar: in 2013–2014, the obesity prevalence in adolescents was 8.4% [29].

Studies of nutritional epidemiology propose that the relationship between diet and the risk of chronic diseases are not caused by the consumption of isolated foods or nutrients. It is believed that the focus on either only food intake or only nutrient intake do not allow for the investigation of the synergistic effect between them in meal composition [30,31]. As foods and drinks are consumed in combination, the studies about dietary patterns including the two allow for the investigation of the whole diet [32], and their use has emerged to consider nutrients interactions and intercorrelations in health outcomes [30,33].

A systematic review of children and adolescent (2–19 years old) nutritional studies from high, medium, and low human development countries identified the presence of unhealthy dietary patterns in 92.5% of the studies included, while 37.5% of them showed traditional dietary patterns [32]. In Brazil, an investigation of adolescents from the PeNSE 2015 edition identified two dietary patterns: an unhealthy pattern based on soft drinks, sweets, fried snacks, and ultra-processed foods; and a healthy pattern, marked by the consumption of beans, fruits, and vegetables [34]. According to other study with larger sample of this database, the prevalence of regular consumption (≥ 5 times/week) of sweets and salty ultra-processed food in these adolescents is higher in females. The soft drinks consumption presented a higher prevalence in males. The consumption of all these food groups was directly associated with school cafeterias [35].

Recent evidence on adolescents suggests the presence of a possible protective effect of traditional or healthy dietary patterns against overweight/obesity [36,37] and a direct association between unhealthy dietary patterns and cardiometabolic risk factors [38]. While several studies have focused on the association between body image and eating behaviors or weight control strategies, there is currently a lack of sufficient evidence on the association between weight misperception and food consumption indicators [9,15,39–43], which denotes the need for further investigation on the subject. Thus, the present study aimed to evaluate the association between weight misperception and dietary patterns in a random sample with national and regional representativeness of Brazilian school adolescents.

## Materials and methods

The present study was conducted in adherence with Strengthening the Reporting of Observational Studies in Epidemiology (STROBE) statement recommendations [44].

### Study design

This cross-sectional study used data from the Brazilian Study of Cardiovascular Risks in Adolescents (ERICA in Portuguese), a multicenter and national school-based survey designed for the estimation of the prevalence of cardiovascular risk factors in adolescents [45,46].

### Setting, study size and participants

Details on the ERICA methodology, including data on the sample, design, participants, and data collection, have been described previously [45–47]. Data were collected from 2013 to 2014, and included those on anthropometric measurements; a self-answered questionnaire, applied through the Personal Digital Assistant (PDA) model LG GM750Q; and a 24-h dietary recall.

The study population comprised adolescents aged 12–17 years from public and private schools located in the urban and rural areas of Brazil’s state capitals and cities with more than 100,000 inhabitants, totaling 273 cities and 102,327 eligible students. The population was further divided into 32 geographic strata and included the 27 state capitals and five units comprising the municipalities of each one of the Brazilian macro-regions. After this, a three-stage cluster sampling plan was adopted. In the first stage, schools were selected using probability proportional to the size, which the size measure of each school was set equal to the ratio between the number of students and the distance from the state capital. The head teachers of the schools selected in the first stage for participation in the study were accordingly engaged. If a head teacher declined the opportunity for their school to participate, their school was replaced by an alternate school from within the same stratum. An alternate school was selected for inclusion based on its similarity to the school it replaced, such as having the same type of administration (public or private), school area (urban or rural), and combination of year levels and class shifts served.

In the second stage, three class shift combinations (morning or afternoon) and form year as a proxy of age (the 7th, 8th and 9th years of elementary school and the 1st, 2nd and 3rd years of high school) were created in each school. One class for each combination was selected in the third stage, and in each selected class, all students were invited to participate in the study.

Adolescents with permanent or temporary physical or mental deficiency, and those who were pregnant were excluded. In this study, only adolescents evaluated as having a normal weight were included.

Although the sampling was comprised of adolescents from urban and rural schools located in the five Brazilian macro-regions, the final sample was not designed to significantly represent the rural adolescent population in Brazil, nor the entire adolescent population of Brazilian macro-regions.

### Variables and measurement

The following data were analyzed in this study: weight underestimation and overestimation (exposures), dietary patterns (outcomes), and individual characteristics (confounders), such as demographic variables (Brazilian macro-region; sex; age group; race/ethnicity; school area; and type of school), poor mental health, and behavioral variables (meals consumed with parents or guardians and excessive screen time).

Weight misperception was defined as the presence of a disagreement between a person’s nutritional status and his/her self-perceived weight status [15]. Height was measured using a potable stadiometer (Alturexata^®^, Minas Gerais, Brazil) and weight measurements were performed by a digital scale (Lider^®^, São Paulo, Brazil); both measurements were evaluated in duplicate. The body mass index (BMI) was calculated as the weight in kilograms divided by the square of the height in meters. Nutritional status was determined by the BMI-for-age z-score according to World Health Organization using different criteria for both sexes [48], and only adolescents with normal weight were considered.

Self-perceived weight status was measured using the questions “Are you satisfied with your weight?” (yes or no) and “In your opinion, at what level is your current weight?” (“below the ideal”, “above the ideal” or “far above the ideal”). The adolescents with normal weight who answered “not satisfied” and “below the ideal” were classified into the underestimation group, and adolescents with normal weight who answered “not satisfied” and “above the ideal” or “far above the ideal” were assigned to the overestimation group; both groups were considered to have weight misperception [42,49].

Dietary patterns were identified from a list with 1.128 food items, obtained with a 24-h dietary recall and collected using the Multiple Pass Method [50]. Further, data on a second 24-h dietary recall were collected in a 10% random subsample of the total sample. The food items were grouped into 20 categories according to similarities in their composition (Table 1). This procedure is commonly employed in this type of analyses and has been used in other studies conducted in the same population [36,51]. The dietary patterns were defined by a *posteriori* method, using principal component factor analysis; patterns were labelled as a result of factors interpretability, according to the food group characteristics [30,31].

**Table 1:** Food groups according to macronutrient related similarities. Study of Cardiovascular Risk in Adolescents (ERICA), Brazil, 2013–2014.

Food groups	Description
Rice	Rice, rice with vegetables, sushi, and rice dishes
Beans	Beans, beans preparations and other pulses
Sugar sweetened beverages	Soft drinks, fruit juices and drinks, milk-based beverages
Corn	Corn, corn flour, “polenta”, other corn preparations
Tubers	Potatoes (except processed potatoes), cassava, yam and flour
Fruits	All fruits
Vegetables	leafy vegetables and legumes
Pasta	Pasta, lasagna, pancakes and pasta preparations
Bread	White and wheat bread, toasts, sandwiches
Cakes/biscuits	Cakes and pies, sweet biscuits, stuffed cookies
Poultry	Poultry and poultry preparations
Meat	Beef, pork and other types of meat, meat preparations
Fish/seafood	Fish and seafood, fish and seafood preparations
Processed meat	Ham, salami, sausage, and others processed meats
Eggs	Eggs and eggs preparations
Milk	Whole and skim milk, yogurts
Cheese	Cheeses, and cheese preparations
Coffee/tea	Coffee, coffee and milk, *cappuccino*, and tea
Desserts/sweets	Pies, sweet pastries, treats, breakfast cereal and cereal bar
Oils and fats	Vegetable oils, butter, animal fat, margarine, heavy cream, sauces, and condiments
Snacks	Pizzas, fast-food, hamburger, deep-fries and baked savory snacks, snacks, cheese bread, French fries, savory biscuits

Demographic variables included macro-region (North; Northeast; Southeast; South or Midwest), sex (boys or girls), age group (12–14 years or 15–17 years), race/ethnicity (white; black or brown, indigenous or Asian), and school area (urban or rural). The type of school (public or private) was used as an indicator of socioeconomic status because of higher proportional spending on education by families of the highest income strata in Brazil [52].

Poor mental health was estimated by the presence of common mental disorders (CMDs), as measured using the 12- item General Health Questionnaire (GHQ-12). The GHQ-12 is a short self-reported screening instrument used to track psychological distress or CMDs, and includes 12 questions, with a 4-point scale [53]. In this study, as in other studies of the same population [54], a cut-off point of 3 or greater was used to confirm CMD presence.

The intake of meals with parents or guardians was estimated using the questions “Does your father (or stepfather) or your mother (or stepmother) or guardian have lunch with you?” and “Does your father (or stepfather) or your mother (or stepmother) or guardian have dinner with you?”. Each question had the following answer options: never or hardly ever; sometimes; almost every day; or every day. This variable was categorized, according to the adolescent’s frequency perception, into: “have lunch and dinner with parents or guardians almost every day or every day”; “have lunch or dinner with parents or guardians almost every day or every day”; and “do not have these meals with parents or guardians”. This variable was used due the positive association between eating meals with parents and a positive body image [55] and the evidence of a protective effect of family meals against risk behaviors in adolescence [56].

Excessive screen time was used as an estimate of sedentary behavior and media exposure time. This variable was measured by the daily time spent using the computer, watching television or playing video games, and according to the relevant recommendations [57] was categorized into greater or lesser than 2 hours per day (yes/no).

### Bias

The introduction of selection and information bias in the ERICA study was prevented by efforts taken in the different phases. A pilot study was conducted across public and private schools in five cities. In the data collection process, anthropometric measurements were performed by trained researchers using appropriate and calibrated equipment, and data were immediately inserted into the PDA. The database was automatically updated using data entered in the PDA and a specific software was used for the collection of 24-h dietary recall data [58]. The data collection was monitored by a central quality control system throughout the study period for the detection of outliers or discrepancies in the measures.

### Statistical methods

The analytical plan of this study was based on a previously defined theoretical model that was developed from a literature review. The prevalence and distribution of the variables investigated were calculated, and the frequencies were estimated with their 95% confidence intervals (95% CIs).

In the definition of the dietary patterns, Kaiser-Meyer-Olkin (KMO) and Bartlett’s sphericity tests were performed for the verification of whether the dietary intake data were suitable for application in the factor analysis. Values greater than 0.5 in the KMO test and p <0.05 in the Bartlett’s test were considered appropriated [59]. Principal component factor analysis was used, followed by varimax orthogonal rotation for the improvement of the factors’ interpretability [30]. Criteria used for factor retention were scree plots, eigenvalues > 1, and factor interpretability. Food groups with a factor loading value ≥0.3 were considered as the most strongly representative of the dietary patterns. Communalities were also evaluated, and a minimum value of 0.10 indicated that the food group sufficiently explained the factor. All dietary patterns were labelled according to the food group characteristics with the highest factor loading values. Additionally, using a “predict” command, a factor score was calculated for each dietary pattern at the individual level [60].

In this analysis, weight misperception categories were the independent variables and dietary patterns, divided into tertiles, were dependent variables. The association between both were presented as prevalence ratios, that were estimated by Poisson regression models. This analytical method was chosen because when the outcome variable has a high frequency, the odds ratio may overestimate the measure of association. In addition, the interpretation of the odds ratio as a risk in this type of study can be misleading, considering that measures of incidence are not measured, rather, prevalence of the studied variables is measured [61].

In the Poisson regression models, the “no misperception” category was the reference group. In simple analysis, we tested each weight misperception category (underestimation and overestimation) with each dietary pattern identified and with covariables of the theoretical model. Only the covariables that presented a p-value <0.20 were maintained in the multiple models. Then, we carried out one adjusted multiple regression model testing the association of each weight misperception category and each one of the dietary patterns, stratified by sex. This resulted in a total of 12 different adjusted models. In the adjusted model, a p-value <0.05 was considered statistically significant.

All statistical analyses were performed in Software for Statistics and Data Science (Stata) [62], version 14.0, using the survey command, with consideration of the natural weights of the sampling design and the use of post-stratification estimators. In these analyses, the sample weight calibration by sex and by each age class was completed in accordance with the recommendations of the ERICA group [45].

### Ethical statements

This study using ERICA data was conducted according to the guidelines laid down in the Declaration of Helsinki. All procedures involving research study participants were approved by the Research Ethics Committees of the Federal University of Rio de Janeiro (protocol number 45/2008), and from all 26 research centers involved. All adolescents interviewed in the ERICA signed written informed consent. For five of the research centers, the participation of students from schools was predicated on written informed consent from their parents or guardians, regardless of the type of participation provided by the volunteer. For the remaining 22 research centers, written informed consent was only necessary in cases where blood collection was required, which did not apply to our study. Informed consent was obtained from the head teachers of all participating schools.

## Results

**Fig 1:**
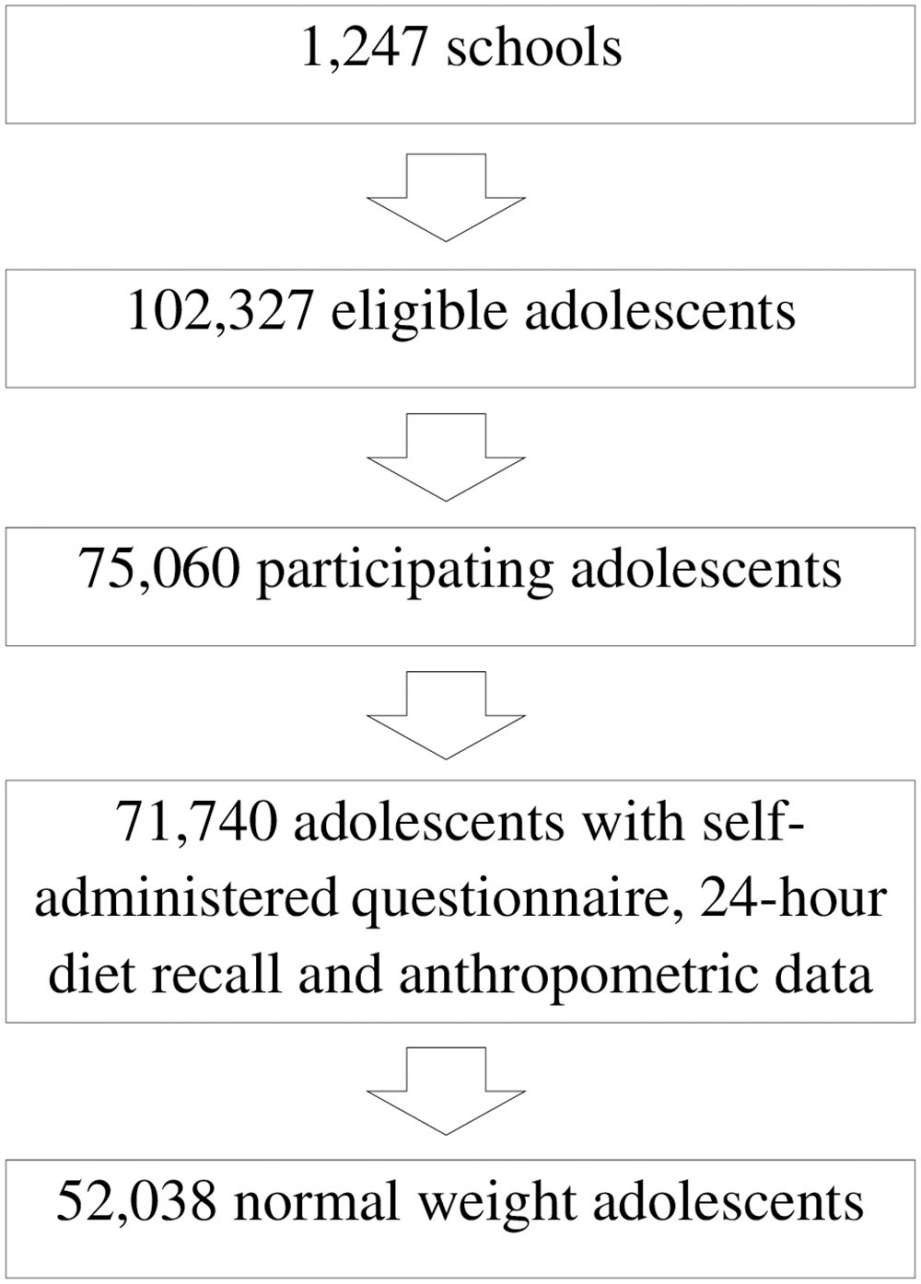
Flow chart showing the enrollment of adolescents with normal weight from the Study of Cardiovascular Risks in Adolescents (ERICA), Brazil, 2013–2014.

A total of 75,060 students from 1,247 schools in 124 municipalities were investigated in the ERICA. Information on participation loss and refusal has been presented previously [47]. Of the participating adolescents, 71,740 had data on anthropometric measurements, as well as answered the self-administered questionnaire and the 24-hour diet recall. Of them, 52,038 who had a normal weight were analyzed in this study (Fig 1).

Table 2 presents the characteristics of the total sample, by sex. The mean age of the adolescents was 14.7 years, and 50.2% were boys. Most of the adolescents were of black or brown race/ethnicity (57.9%, 95% CI: 56.2, 59.5), and studied in public schools (83.6%, 95% CI: 79.2, 87.3) and those located in urban areas (95.4%, 95% CI: 85.7, 98.6), with no sex-related differences. The prevalence of CMDs was greater in the girls (38.4%, 95% CI: 36.7, 39.9] than boys (25.6%, 95% CI: 24.5, 26.6). The prevalence of excessive screen time was 56.7% (95% CI:55.6, 58.3), with the absence of sex-related differences. Conversely, the prevalence of the intake of lunch and dinner with parents or guardians was higher in the boys (51.8%, 95% CI: 50.0, 53.5) than girls (42.6%, 95% CI: 41.2, 44.0).

**Table 2:** Sociodemographic characteristics, mental health, behaviors and weight misperception in participants with normal weight of the Study of Cardiovascular Risks in Adolescents (ERICA), Brazil, 2013–2014.

	Girls (n = 29,325)		Boys (n = 22,713)		Total (n = 52,038)	
	%	95% CI	%	95% CI	%	95% CI
**Age group**						
12–14 years	52.5	**	52.9	**	52.7	**
15–17 years	47.5	**	47.1	**	47.3	**
**Race/ethnicity**						
White	39.2	37.3, 41.1	39.2	37.2, 41.2	39.2	37.5, 40.9
Black or brown	58.0	56.2, 59.9	57.7	55.7, 59.7	57.9	56.2, 59.5
Indigenous or Asian	2.8	2.4, 3.2	3.1	2.6, 3.6	2.9	2.6, 3.3
**Macro-region**						
North	8.5	**	8.4	**	8.4	**
Northeast	21.4	**	21.2	**	21.3	**
Southeast	50.6	**	50.9	**	50.8	**
South	11.8	**	11.8	**	11.8	**
Midwest	7.7	**	7.6	**	7.7	**
**Type of school**						
Public	83.6	78.9, 87.3	83.7	79.3, 87.4	83.6	79.2, 87.3
Private	16.4	12.7, 21.1	16.3	12.6, 20.8	16.4	12.7, 20.8
**School area**						
Urban	95.6	86.1, 98.7	95.1	85.2, 98.5	95.4	85.7, 98.6
Rural	4.3	1.3, 13.9	4.9	1.5, 14.8	4.6	1.4, 14.3
**Common mental disease** *	38.4^a^	36.7, 39.9	21.5	20.1, 22.9	29.9	28.8, 31.0
**Screen time > 2 hours/day**	56.0	54.6, 57.2	57.4	54.5, 60.2	56.7	55.6, 58.3
**Meals with parents almost everyday or everyday**						
Lunch and dinner	42.6	41.2, 44.0	51.8^a^	50.0, 53.5	47.2	45.9, 48.6
Lunch or dinner	22.7^a^	21.6, 23.8	19.4	17.9, 20.9	21.0	20.1, 22.0
Don’t have these meals with their parents	34.7^a^	33.3, 36.2	28.9	27.7, 30.1	31.8	30.8, 32.8
**Weight misperception**						
No misperception	57.4	55.9, 58.9	74.5^a^	73.4, 75.6	66.0	65.0, 66.9
Underestimation	16.9	16.0, 17.8	17.3	16.4, 18.1	17.1	16.5, 17.7
Overestimation	25.7^a^	24.2, 27.4	8.2	7.4, 9.1	17.0	16.0, 18.0

*Measured by 12- item General Health Questionnaire > = 3.

** Variables used to calculate the natural weights and calibration factors of the sample.

^a^ P value < 0.05.

Regarding body image, 34.0% (95% CI: 33.0, 35.0) misperceived their weight. This prevalence was even greater among girls (42.6%, 95% CI: 41.1, 44.1) than boys (25.6%, 95% CI: 24.5, 26.6). The girls also showed a higher prevalence of weight overestimation (25.7%, 95% CI: 24.2, 27.2) than boys (8.2%, 95% CI: 7.4, 9.1). Given the differences identified in the levels of overestimation between the boys and girls, separate analyses for each sex were performed.

Table 3 presents the three dietary patterns identified, explaining 21.6% of the variance in the dietary intake in this population. A value of 0.53 in the KMO test and p>0.05 in Bartlett’s sphericity tests were obtained.

**Table 3:** Factor loadings values† of the identified dietary patterns of Brazilian adolescents with normal weight. Study of Cardiovascular Risk in Adolescents (ERICA), Brazil, 2013–2014.

Food Groups	Traditional Brazilian	Processed meat sandwiches and coffee	Ultra-processed and sweet	Communality (%)
Rice	**0.7619**	0.0423	-0.0093	58.2
Beans	**0.6728**	0.1118	0.0022	46.5
Fruits	0.0831	-0.0162	0.1635	3.4
Corn	-0.0088	0.1800	-0.0256	3.3
Vegetables	**0.3332**	-0.0719	0.2398	17.4
Tubers	0.1883	-0.0053	0.2365	9.1
Pasta	-0.2390	0.2309	0.2654	18.1
Poultry	0.1825	0.1261	0.0374	5.1
Meat	**0.4762**	-0.0929	0.1907	27.2
Fish/seafood	0.0300	-0.0086	0.0206	0.1
Processed meat	-0.0193	**0.3365**	0.1186	12.8
Eggs	0.0647	0.1973	0.0465	4.5
SSB*	0.1193	0.0683	**0.6889**	49.3
Desserts/sweets	-0.0301	-0.0201	**0.4240**	18.1
Coffee/tea	0.1697	**0.4813**	-0.2590	32.7
Bread	0.0812	**0.7047**	-0.0002	50.3
Cakes/biscuits	0.0374	0.0480	0.2956	9.1
Milk/yogurt	0.0154	0.0713	0.2950	9.3
Cheese	-0.1067	**0.4253**	0.2544	25.7
Oils and fats	0.0188	**0.4927**	0.0803	25.0
Snacks	-0.1533	-0.1668	**0.4910**	29.2
Eigenvalues	1.60	1.47	1.47	
% of explained variance	7.6	7.0	7.0	
% of accumulated explained variance	7.6	14.6	21.6	

* SSB, Sugar sweetened beverages.

^†^ Highlighted values are food groups that presented a factor loading ≥ 0.3, which indicate a high correlation between the food group and the dietary pattern.

The first dietary pattern presented factor loading values greater than 0.3 for rice, beans, vegetables and meat, and was labelled “Traditional Brazilian”. The second dietary pattern comprised processed meat, coffee/tea, bread, cheese, oils and fats, and was labelled “Processed meat sandwiches and coffee”. The last pattern presented the highest factor loading values for sugar-sweetened beverages, desserts/sweets and ultra-processed foods, and was labelled “Ultra-processed and sweet foods”. Boys presented the highest terciles of all the dietary patterns (Table 4).

**Table 4:** Dietary pattern terciles in participants with normal weight of the Study of Cardiovascular Risks in Adolescents (ERICA), Brazil, 2013–2014.

	Girls (n = 29,325)		Boys (n = 22,713)		Total (n = 52,038)		P-value
	%	95% CI	%	95% CI	%	95% CI	
**“Traditional Brazilian” dietary pattern**							
1st tercile	38.0	**35.8, 40.2**	24.2	22.5, 25.9	31.1	29.3, 32.9	<0.001
2st tercile	34.5	**33.3, 35.7**	30.7	29.0, 32.4	32.6	31.4, 33.7	
3st tercile	27.5	24.9, 30.3	45.2	**42.3, 48.1**	36.4	33.8, 39.0	
**“Processed meat sandwiches and coffee” dietary pattern**							
1st tercile	39.2	**37.7, 40.8**	28.0	26.5, 29.6	33.6	32.3, 35.0	<0.001
2st tercile	34.6	**33.1, 36.1**	31.3	29.8, 32.8	32.9	31.8, 34.0	
3st tercile	26.2	24.7, 27.7	40.7	**38.7, 42.4**	33.5	32.2, 34.7	
**“Ultra-processed and sweet foods” dietary pattern**							
1st tercile	33.3	31.3, 35.3	34.1	32.2, 36.1	33.7	32.1, 35.3	<0.001
2st tercile	36.2	**34.6, 37.9**	31.4	30.2, 32.7	33.8	32.9, 34.8	
3st tercile	30.5	29.2, 31.7	34.4	**32.6, 36.3**	32.4	31.2, 33.8	

In the analysis of the level of weight underestimation in the girls, age group, race/ethnicity, macro-region, type of school, presence of CMD, intake of meals with parents, and excessive screen time were selected for the multiple model. For overestimation, the variables selected were: age group, race/ethnicity, macro-region, type of school, school area, CMD, excessive screen time, and intake of meals with parents. Among boys, age group, macro-region, type of school, school area, presence of CMD, and intake of meals with parents were used in the multiple model for underestimation. Overestimation models were adjusted for the type of school, presence of CMD, excessive screen time, and intake of meals with parents (Table 5).

**Table 5:** Unadjusted prevalence ratio of the underestimation and overestimation of body weight, by sex, according to participants’ sociodemographic characteristics, mental health, family meals, and dietary patterns in adolescents with normal weight of Study of Cardiovascular Risks in Adolescents (ERICA), Brazil, 2013–2014.

	Underestimation						Overestimation					
	Girls			Boys			Girls			Boys		
	PR*	95% CI	P value	PR*	95% CI	P value	PR*	95% CI	P value	PR*	95% CI	P value
**Age group**			<0.001^a^			<0.001^a^			0.04^a^			0.26
12–14 years	--	--		--	--		--	--		--	--	
15–17 years	1.25	1.13, 1.38		1.59	1.42, 1.79		1.13	1.01, 1.29		1.13	0.92, 1.39	
**Race/ethnicity**			0.13^a^			0.59			<0.01^a^			0.94
White	--	--		--	--		--	--		--	--	
Black or brown	1.10	0.99, 1.20		1.03	0.89, 1.18		0.84	0.76, 0.93		0.99	0.79, 1.24	
Indigenous or Asian	1.16	0.92, 1.47		1.15	0.88, 1.50		0.83	0.66, 1.04		0.91	0.54, 1.53	
**Macro-region**			<0.001^a^			<0.001^a^			<0.001^a^			0.63
North	--	--		--	--		--	--		--	--	
Northeast	0.94	0.84, 1.05		1.06	0.95, 1.16		1.03	0.92, 1.16		0.93	0.75, 1.14	
Southeast	0.65	0.58, 0.73		0.69	0.62, 0.78		1.28	1.12, 1.46		1.10	0.89, 1.37	
South	0.65	0.56, 0.76		0.70	0.56, 0.87		1.56	1.38, 1.76		1.01	0.77, 1.33	
Midwest	0.71	0.62, 0.81		0.84	0.74, 0.96		1.40	1.27, 1.54		1.06	0.84, 1.34	
**Type of school**			<0.01^a^			0.04^a^			<0.001^a^			<0.01^a^
Public	--	--		--	--		--	--		--	--	
Private	0.76	0.65, 0.89		1.14	1.00, 1.30		1.39	1.22, 1.57		1.38	1.15, 1.65	
**School area**			0.94			0.10^a^			0.04^a^			0.23
Urban	--	--		--	--		--	--		--	--	
Rural	0.99	0.76, 1.29		0.76	0.55, 1.05		0.83	0.68, 0.99		0.84	0.62, 1.12	
**Common mental disease (GHQ12 > = 3)**			<0.001^a^			<0.001^a^			<0.001^a^			<0.001^a^
No	--	--		--	--		--	--		--	--	
Yes	1.47	1.31, 1.66		1.42	1.20, 1.68		1.51	1.38, 1.66		1.77	1.39, 2.27	
**Screen time > 2 hours/day**			<0.001^a^			0.55			0.08^a^			0.02^a^
No	--	--		--	--		--	--		--	--	
Yes	1.28	1.14, 1.43		0.96	0.84, 1.10		1.12	0.98, 1.26		1.24	1.04, 1.48	
**Meals with parents almost everyday or everyday**			<0.001^a^			<0.001^a^			<0.001^a^			<0.01^a^
Lunch and dinner	--	--		--	--		--	--		--	--	
Lunch or dinner	1.22	1.04, 1.44		1.20	1.01, 1.42		1.24	1.10, 1.39		1.43	1.12, 1.82	
Don’t have these meals with their parents	1.31	1.17, 1.45		1.40	1.20, 1.59		1.27	1.16, 1.40		1.30	1.03, 1.63	
**“Traditional Brazilian” dietary pattern**			0.02^a^			0.62			<0.001^a^			<0.01^a^
1st tercile	--	--		--	--		--	--		--	--	
2st tercile	1.11	0.94, 1.32		0.97	0.82, 1.16		0.79	0.73, 0.86		0.92	0.72, 1.17	
3st tercile	1.22	1.05, 1.41		1.04	0.90, 1.20		0.69	0.61, 0.77		0.70	0.56, 0.88	
**“Processed meat sandwiches and coffee” dietary pattern**			0.12^a^			0.02^a^			<0.01^a^			0.44
1st tercile	--	--		--	--		--	--		--	--	
2st tercile	1.07	0.93, 1.22		1.00	0.86, 1.18		0.99	0.91, 1.10		0.86	0.68, 1.09	
3st tercile	1.14	0.99, 1.31		1.20	1.04, 1.38		0.79	0.70, 0.89		0.89	0.69, 1.16	
**“Ultra-processed and sweet foods” dietary pattern**			<0.01^a^			<0.01^a^			0.09^a^			0.49
1st tercile	--	--		--	--		--	--		--	--	
2st tercile	1.06	0.91, 1.23		1.05	0.89, 1.24		1.04	0.91,1.18		1.16	0.88, 1.54	
3st tercile	1.29	1.10, 1.51		1.31	1.12, 1.52		0.93	0.85, 1.02		1.04	0.79, 1.37	

*Prevalence ratio.

^†^ GHQ12: 12- item General Health Questionnaire.

^a^ P-value < 0.20.

The results of the adjusted analysis of the association between weight misperception and dietary patterns, by sex, are presented in Table 6. For girls, weight underestimation was directly associated with the highest tertiles of “Traditional Brazilian” (prevalence ratio [PR]: 1.24; 95% CI: 1.08, 1.43) and “Ultra-processed and sweet foods” dietary patterns (PR: 1.29; 95% CI: 1.09, 1.54). Weight overestimation was inversely associated with the highest tertiles of the “Traditional Brazilian” (PR: 0.74; 95% CI: 0.66, 0.83), “Processed meat sandwiches and coffee” (PR: 0.83; 95% CI: 0.73, 0.94) and “Ultra-processed and sweet foods” (PR: 0.87; 95% CI: 0.78, 0.96) dietary patterns.

**Table 6:** Adjusted prevalence ratio of the underestimation and overestimation of body weight, by sex, according to the dietary patterns in participants with normal weight of the Study of Cardiovascular Risks in Adolescents, Brazil, 2013–2014.

	Underestimation				Overestimation			
	Girls†		Boys‡		Girls§		Boys¶	
	PR*	95% CI	PR*	95% CI	PR*	95% CI	PR*	95% CI
**“Traditional Brazilian” dietary pattern**								
1st tercile	**--**	**--**	**--**	**--**	**--**	**--**	**--**	**--**
2st tercile	1.06	0.89, 1.26	1.00	0.84, 1.19	0.82 ^a^	0.74, 0.90	1.03	0.80, 1.31
3st tercile	1.24^a^	1.08, 1.43	1.07	0.92, 1.24	0.74 ^a^	0.66, 0.83	0.79 ^a^	0.63, 0.99
**“Processed meat sandwiches and coffee” dietary pattern**								
1st tercile	**--**	**--**	**--**	**--**	**--**	**--**	**--**	**--**
2st tercile	1.06	0.91, 1.23	1.01	0.87, 1.18	1.00	0.91, 1.10	0.90	0.71, 1.14
3st tercile	1.08	0.95, 1.23	1.16 ^a^	1.01, 1.34	0.83 ^a^	0.73, 0.94	0.97	0.74, 1.27
**“Ultra-processed and sweet foods” dietary pattern**								
1st tercile	**--**	**--**	**--**	**--**	**--**	**--**	**--**	**--**
2st tercile	1.08	0.92, 1.27	1.06	0.91, 1.25	0.97	0.87, 1.09	1.05	0.77, 1.43
3st tercile	1.29 ^a^	1.09, 1.54	1.29 ^a^	1.10, 1.51	0.87 ^a^	0.78, 0.96	0.87	0.65, 1.16

*Prevalence ratio.

^†^ Adjusted by common mental disease, age group, meals with parents or guardians, screen time, macro-region, type of school and race/ethnicity.

^‡^ Adjusted by common mental disease, age group, meals with parents or guardians, type of school, macro-region and school area.

^§^Adjusted by common mental disease, meals with parents or guardians, macro-region, type of school, race/ethnicity, age group, school area and screen time.

^¶^ Adjusted by common mental disease, type of school, meals with parents or guardians and screen time.

^a^ P-value < 0.05.

In boys, a direct association was observed between weight underestimation and greater adherence to “Processed meat sandwiches and coffee” (PR: 1.16; 95% CI: 1.01, 1.34) and “Ultra-processed and sweet foods” (PR: 1.29; 95% CI: 1.10, 1.51) dietary patterns. Weight overestimation was inversely associated with the highest tertile of the “Traditional Brazilian” dietary pattern (PR: 0.79; 95% CI: 0.63, 0.99] (Table 6).

## Discussion

This study, to the best of our knowledge, is the first to analyze the association between weight misperception and dietary patterns in a representative sample in Brazil. Approximately one-third of adolescents were found to have weight misperception, with a higher overestimation prevalence noted in the girls. Three dietary patterns were identified in association with this misperception: “Traditional Brazilian” (rice, beans, vegetables and meat), “Processed meat sandwiches and coffee” (processed meat, coffee/tea, bread, cheese, oils and fats) and “Ultra-processed and sweet foods” (sugar-sweetened beverages, desserts/sweets and ultra-processed foods). A direct association was observed between weight underestimation and the “Ultra-processed and sweet foods” pattern, and an inverse association was noted between weight overestimation and the “Traditional Brazilian” dietary pattern in both sexes. Among girls, an inverse association was observed between overestimation and all three patterns identified.

The direct association identified between underestimation and the “Traditional Brazilian” pattern is beneficial due to the protective effect of this type of dietary pattern against abdominal obesity [63–65]. However, the association with the “Processed meat sandwiches and coffee” pattern in boys, and “Ultra-processed and sweet foods” pattern in both sexes may be indicative of a low recurrence of weight gain-related concerns among adolescents with weight underestimation, as already evidenced in another study [66].

In contrast, the inverse association noted between weight overestimation and all the three identified dietary patterns in girls presumably evince an apprehensiveness of eating in this population. Although this hypothesis cannot be confirmed, as our study did not investigate the factors related to eating disorders, the inverse relationship observed with the “Traditional Brazilian” pattern indicates that this behavior may be harmful. According to evidence in South Korea, girls with weight overestimation tend to have poor eating habits, to employ unhealthy dieting methods for weight loss, and do not have appropriate eating habits for the achievement of optimal health and weight control [23]. In a recent study in Brazil, adolescents who self-perceived as overweight showed a 2.381-fold higher prevalence of unhealthy weight control behaviors, such as skipping meals, using shakes or supplements as food substitutes, smoking cigarettes, or taking diet pills. The odds of disordered eating behaviors, such as binge-eating episodes, diuretics and laxatives use, self-induced vomiting, or extremely strict diet or fasting, was 1.795-fold higher in adolescents with overweight perceived status compared to adolescents who self-perceived as normal weight [67]. These results corroborate the evidence that, despite greater attempts to lose weight, adolescents of normal weight who self-perceive as overweight may not effectively translate their intentions into healthy weight loss behaviors [18].

The prevalence of weight misperception found in this investigation was similar to that found among other studies of Brazilian adolescents [68,69]. Although the aforementioned studies also included adolescents who were underweight and overweight, an investigation conducted in Salvador, a city of Brazil, showed that girls with normal weight were most likely to overestimate their body weight [69]. The ability to compare this prevalence with other Latin American countries is limited, as studies with body image perception or (dis)satisfaction are more common in these countries. In a study carried out in seven South American cities to establish the reliability and validity of instruments to measure body image, researchers found that 15.4% of normal weight adolescents underestimated their weight and 11.5% overestimated their weight [70]. In other territories, the rates of weight underestimation and overestimation ranged between 12.4% and 27.0% in China [71], 17.8% and 9.8% in Italy [72], 22.8% and 15.7% in South Korea [9], 29.1% and 11.6% in the United States [42], and 30.8% and 28.4% in Iran [39].

In the present study, only adolescents with normal weight were analyzed. This decision was made considering the different aspects of individuals who are underweight and overweight. Weight underestimation in overweight individuals, for example, can be associated with the lack of motivation to adopt healthy habits and increasing of the risk for developing non-communicable disease (NCD) [73], which would expand the scope of discussion of this study. Furthermore, the evidence indicates that adolescents with normal weight are likelier to misperceive their weight [43]. It should be noted that each of the aforementioned studies used different instruments for the measurement of the level of weight misperception, and the presence of methodological diversity across the surveys cannot be ruled out [74,75]. Therefore, the obtained weight misperception prevalence rates should be carefully compared.

Another aspect to be considered is that the traditional forms of body image distortion, namely underestimation and overestimation, were considered separately due the differing etiologies and outcomes. Like in the case of other investigations [9,15,40,49,73], in this study, girls showed a higher prevalence of weight overestimation. However, unlike in most previous surveys, in which boys were found to underestimate their body weight to a greater magnitude [9,40,41,71], no differences in the level of underestimation between the sexes were observed in the present analysis.

This higher prevalence of weight overestimation in girls may be influenced by social constructs in relation to body image: contemporary female beauty is often associated with thinness; thus, weight misperception in girls may be related to the expectation of a social ideal of a perfect body [76]. The internalization of a thin ideal and the comparisons with models, celebrities, and peers are positively correlated with body image concerns in adolescent girls. This body comparison with peers and models seems to mediate the relationship between the endorsement of norms for thinness and body image concerns in this group [77]. The use of the internet and social media also influences this issue. According to a study of Australian girls who were 13–15 years old, internet exposure was associated with internalization of the thin ideal and body surveillance. Further, the use of Facebook was correlated to higher body concerns in this group [7].

The dietary patterns identified in this study were labelled according to their food group characteristics. The first dietary pattern was termed “Traditional Brazilian” as it comprised foods traditionally consumed by the Brazilian population [78]. The second pattern, comprising foods usually consumed between meals or those that replace lunch and dinner, such as sandwiches or hot dogs in Brazil [79], was labelled “Processed meat sandwiches and coffee”. The last pattern was labelled “Ultra-processed and sweet foods” and comprised foods that are generally highly processed and well-accepted by adolescents [80].

When comparing the results of this study with the results of investigations with analogous methodology for extracting dietary patterns, we found that the composition of “Traditional Brazilian” dietary patterns was similar to the namesake pattern identified in other studies of Brazilian adolescents [81–83], as well as the “Common Brazilian” pattern identified between adolescents of Fortaleza, Brazil [84]. All these dietary patterns had rice, beans, and meats in common; however, there were some differences in the studies related to the presence of processed meats [81], vegetables [81,82], fruit juice [81] and pasta [84].

The “Processed meat sandwiches and coffee” pattern, in turn, was comparable to the composition of “Bread and Butter” [81], “Snacks pattern” [82,83], and “Coffee and bread” [84] dietary patterns identified in other studies with Brazilian adolescents. Bread, coffee and fats were present in all these dietary patterns, and processed meat and cheese were presented in some of patterns [82,83].

Regarding the “Ultra-processed and sweet foods” dietary pattern, the composition that we found was remarkably similar to the “Western pattern” identified in João Pessoa, Brazil [83]; and to the “Fast Food Pattern” of the Brazilian Household Budget Survey 2008–2009 [82]. Both patterns are characterized by the presence of sweetened beverages, sweets and desserts, and snacks like pizza, deep-fries, and baked savory foods.

According to the NOVA (non-acronym name) food classification, ultra-processed foods are formulations of ingredients based on industrial procedures, which are produced with the objective to create durable, accessible, convenient, attractive, ready-to-eat or ready-to-heat products. They are formulated to be extremely palatable; most of them are energy-dense and rich in added sugar, saturated fats, and sodium, and have low dietary fiber levels. During the processing stage, these foods are treated with artificial or chemical additives [26,85]. In adults, the intake of this type of food is related to an increased risk of cancer and cardiovascular disease [86,87]. Adolescents can also be harmed by the consumption of ultra-processed foods: studies show evidence of a direct association of ultra-processed foods with metabolic syndrome [88] and body fat [89].

The Food Guide for Brazilian Population recommends that people’s dietary patterns include the intake of a variety of unprocessed or minimally processed foods, such as grains, legumes, vegetables, fruits, nuts, milk, meat and other foods [90], some of which were present in the “Traditional Brazilian” pattern. The intake of ultra-processed foods, such as those reported in the “Ultra-processed and sweet foods” pattern, should be avoided.

The direct association observed between weight underestimation and the “Ultra-processed and sweet foods” pattern, and the inverse association between weight overestimation and the “Traditional Brazilian” dietary pattern in this study corroborates the hypothesis that weight misperception may be related to poor eating habits in adolescents, as found in other countries. Underestimation was shown to be directly associated with the insufficient consumption of fruits and vegetables in Iran [39] and sugar-sweetened beverages in Canada [91]. Moreover, an inverse association between overestimation and Mediterranean-style eating patterns was observed in Italian adolescents [72]. In Japan, a direct association was observed between overestimation and snack consumption between in the adolescent population [41]. However, despite the favorable evidence, caution is needed when generalizing these associations. In another study of Brazilian adolescents in 2009, no associations were identified between weight misperception and adherence to dietary patterns. It is noteworthy that the dietary patterns found in the Andrade et al. [69] study were identified by a different methodology than the one used in the present investigation, which limits the comparability of the results.

It is difficult to compare the association we observed between weight misperception and dietary patterns with that noted in other studies. This limitation is due to the lack of sufficient data obtained from association studies involving other dietary markers, such as energy intake, food consumption and eating behaviors [9,39,40,43]. Moreover, the use of different methods for the recording, consolidation and analyses of food consumption indicates that caution must be exercised in the comparison of the results.

Furthermore, studies focusing on the association between body image and food consumption present conflicting results, denoting the lack of a consensus in the scientific literature on this theme. In South Korea, for example, adolescents who underestimated their weight presented a higher frequency of soda consumption (considered a negative indicator) and breakfast intake (a positive behavior) [9]. Another study, conversely, showed the presence of an inverse association between weight underestimation and breakfast intake, and a direct association between overestimation and daily soda consumption [15].

Considering the adverse associations identified in the present study, health policymakers should focus on the implementation of intervention programs aimed at guiding children and adolescents in the accurate perception of their body weight. School programs focused on media literacy, self-esteem, and the influence of peers have been shown to be effective in improving body image, although the size effect is small [92]. The use of disclaimers as labels in advertisements and photos with thin-ideal images has been proposed for the protection of young women’s body image and mood; however, this strategy has been proven ineffective [93,94].

The ERICA comprises a large sample; therefore, our study, which used data from that survey, makes important contributions in the context of body image and dietary patterns. The methodologic rigor employed in the sample design, data collection, and databank analysis reinforce the robustness of the present study. Additionally, the weight and height measurements in the whole sample, performed by trained evaluators using validated equipment, increase the reliability of the observed nutritional status.

This investigation, however, also has some limitations that must be considered. First, owing to its cross-sectional design, no causal inferences could be made, and only associations between weight misperception and dietary pattern were identified. Second, only school-going adolescents with normal weight were included; therefore, the results cannot be generalized to the entire Brazilian adolescent population. Third, data on the second 24-h dietary recall were collected only in two students per class, which can be insufficient to estimate intra-individual variability. Fourth, although the self-answered questionnaire is advantageous for avoiding interference from the interviewer, it can lead to response bias, as the participant may not understand the questions presented or may endeavor to give answers that are socially acceptable. Finally, the body weight perception in the present study was assessed using a single question, and, as this factor is multidimensional, the measurement method may not be sufficient for the reflection of the actual body image in its complexity.

## Conclusions

Our findings indicate the presence of a high prevalence of weight misperceptions in Brazilian adolescents with normal weight, with the overestimation prevalence shown to be higher in girls. The inverse association observed between overestimation and the “Traditional Brazilian” dietary pattern, as well as the direct association noted between underestimation and the “Ultra-processed and sweet foods” pattern in both sexes indicates that weight misperception is related to unhealthy eating habits in adolescents. Programs aimed at improving body image in adolescents can be useful in correcting the aforementioned misperceptions, and consequently, in promoting better eating habits and contributing to an improved general health status in this population. To expand upon the findings of this study, future research should include longitudinal studies to assess the impact of weight misperception on dietary patterns and nutritional status of adolescents.

## Supporting information

S1 File (PDF)

S1 Dataset (DTA)
