# Supplementary material for: Association between body weight misperception and dietary patterns in Brazilian adolescents: Cross-sectional study using ERICA data
Source: PLoS One. 2021 Sep 23;16(9):e0257603. doi: 10.1371/journal.pone.0257603 (PMC8460023; doi:10.1371/journal.pone.0257603)
Supplement: S1 File — (PDF) [file pone.0257603.s001.pdf]

Association between body weight misperception and dietary patterns in Brazilian adolescents: cross-sectional study using ERICA data

Simoni Urbano da Silva[1]*, Mariane de Almeida Alves[2], Francisco de Assis Guedes de Vasconcelos[3], Vivian Siqueira Santos Gonçalves[1], Laura Augusta Barufaldi[4], Kenia Mara Baiocchi de Carvalho[1]

[1] Graduate Program of Public Health, Faculty of Health Sciences, University of Brasilia, Brasilia, DF, Brazil.

[2] Department of Nutrition, School of Public Health, University of Sao Paulo, SP, Brazil.

[3] Graduate Program in Nutrition, Federal University of Santa Catarina, Florianópolis, SC, Brazil.

[4] Population Research Division, Brazilian National Cancer Institute José Alencar Gomes da Silva, Rio de Janeiro, RJ, Brazil.

Dictionary of dataset variables used

**Variable**	**Label**	**Code**
cod_UPA	Primary sampling unit code	Sample weighting variables
cod_stra_sel	Sample selection stratum code	Sample weighting variables
natweight	Natural weight of the design that represents the probability of inclusion in the sample	Sample weighting variables
sampleweight	Calibrated sample weight	Sample weighting variables
poststrat	Post-stratum	Post-stratification variables
postweight	Post-weight	Post-stratification variables
id_erica	Participant identification number	Continuous variable
weightsatisfaction	Student's satisfaction with their own weight	1 = Yes<br>2 = No
currentweight	Opinion about current weight	1 = Below the ideal<br>2 = Above the ideal

		3 = Far above the ideal
weightkg	Weight in kilograms	Continuous variable
height1	1st height measurement in centimeters	Continuous variable
height2	2st height measurement in centimeters	Continuous variable
heightaverage	Average between the two height measurements	Continuous variable
bmi	Body Mass Index in kg/m²	Continuous variable
zescore_bmi	Z-score of BMI-for-age	Continuous variable
nutritionalstatus	Nutritional status classification, according to BMI-for-age z-scores (WHO, 2007)	3 = Normal weight*<br><br>*In this study, only adolescents with normal weight were analyzed
weightmisperception	Presence of weight misperception	0 = No<br>1 = Yes
underestimation	Presence of weight underestimation	0 = No<br>1 = Yes
overestimation	Presence of weight overestimation	0 = No<br>1 = Yes
sex	Sex	0 = Female<br>1 = Male
macroregion	Brazilian macro-region	0 = North<br>1 = Northeast<br>2 = Southeast<br>3 = South<br>4 = Midwest
typeschool	Type of school	0 = Public<br>1 = Private
schoolarea	School area	0 = Urban

		1 = Rural
agegroup	Age group	0 = 12 -14 years old<br>1 = 15 -17 years old
ethnicity	Race/ethnicity	0 = White<br>1 = Black or brown<br>2 = Indigenous or Asian
cmd	Presence of common mental disease (according GHQ12 >=3)	0 = No<br>1 = Yes
lunchparents	Frequency of having lunch with parents or guardians	1 = Never or hardly ever<br>2 = Sometimes<br>3 = Almost every day<br>4 = Every day
dinnerparents	Frequency of having dinner with parents or guardians	1 = Never or hardly ever<br>2 = Sometimes<br>3 = Almost every day<br>4 = Every day
mealsparents	To have meals with parents or guardians almost everyday or everyday	0 = Lunch AND dinner<br>1 = Lunch OR dinner<br>2 = Do not have meals with parents almost everyday or everyday
screentime	Daily time spent using the computer, watching television or playing video games	1 = Do not do these activities on a weekday<br>2 = Less than 1 hour a day<br>3 = About 1 hour a day<br>4 = About 2 hours a day<br>5 = About 3 hours a day<br>6 = About 4 hours a day<br>7 = About 5 hours a day<br>8 = About 6 hours a day<br>9 = About 7 or more hours a day

		77 = Do not know/do not remember
catscreentime	Screen time > 2 hours/day	0 = No<br>1 = Yes
ricegroup	Amount in grams of the consumption of the “Rice” food group	Continuous variable
beansgroup	Amount in grams of the consumption of the “Beans” food group	Continuous variable
ssbgroup	Amount in grams of the consumption of the “Sugar sweetened beverages” food group	Continuous variable
corngroup	Amount in grams of the consumption of the “Corn” food group	Continuous variable
tubersgroup	Amount in grams of the consumption of the “Tubers” food group	Continuous variable
fruitsgroup	Amount in grams of the consumption of the “Fruits” food group	Continuous variable
veggroup	Amount in grams of the consumption of the “Vegetables” food group	Continuous variable
pastagroup	Amount in grams of the consumption of the “Pasta” food group	Continuous variable
breadgroup	Amount in grams of the consumption of the “Bread” food group	Continuous variable

cakegroup	Amount in grams of the consumption of the “Cakes/biscuits” food group	Continuous variable
poultrygroup	Amount in grams of the consumption of the “Poultry” food group	Continuous variable
meatgroup	Amount in grams of the consumption of the “Meat” food group	Continuous variable
fishgroup	Amount in grams of the consumption of the “Fish/seafood” food group	Continuous variable
processedmeatgroup	Amount in grams of the consumption of the “Processed meat” food group	Continuous variable
eggsgroup	Amount in grams of the consumption of the “Eggs” food group	Continuous variable
milkgroup	Amount in grams of the consumption of the “Milk” food group	Continuous variable
cheesegroup	Amount in grams of the consumption of the “Cheese” food group	Continuous variable
coffeegroup	Amount in grams of the consumption of the “Coffee/tea” food group	Continuous variable
sweetgroup	Amount in grams of the consumption of the “Desserts/sweets” food group	Continuous variable
oilsgroup	Amount in grams of the consumption of the “Oils and fats” food group	Continuous variable

snacksgroup	Amount in grams of the consumption of the “Snacks” food group	Continuous variable
p1	Individual factor scores of “Traditional Brazilian” dietary pattern	Continuous variable
p2	Individual factor scores of “Processed meat sandwiches and coffee” dietary pattern	Continuous variable
p3	Individual factor scores of “ultra-processed and sweet” dietary pattern	Continuous variable
catp1	3 quantiles of p1	1 = 1st tertile<br>2 = 2nd tertile<br>3 = 3rd tertile
catp2	3 quantiles of p2	1 = 1st tertile<br>2 = 2nd tertile<br>3 = 3rd tertile
catp3	3 quantiles of p3	1 = 1st tertile<br>2 = 2nd tertile<br>3 = 3rd tertile
